# Lack of Cetuximab induced skin toxicity in a previously irradiated field: case report and review of the literature

**DOI:** 10.1186/1748-717X-5-38

**Published:** 2010-05-17

**Authors:** Madhava R Kanakamedala, Satyaseelan Packianathan, Srinivasan Vijayakumar

**Affiliations:** 1 Department of Radiation Oncology, University of Mississippi Medical Center, 350 W Woodrow Wilson Drive #1600, Jackson, MS 39213, USA

## Abstract

**Introduction:**

Mutation, amplification or dysregulation of the EGFR family leads to uncontrolled division and predisposes to cancer. Inhibiting the EGFR represents a form of targeted cancer therapy.

**Case report:**

We report the case of 79 year old gentlemen with a history of skin cancer involving the left ear who had radiation and surgical excision. He had presented with recurrent lymph node in the left upper neck. We treated him with radiation therapy concurrently with Cetuximab. He developed a skin rash over the face and neck area two weeks after starting Cetuximab, which however spared the previously irradiated area.

**Conclusion:**

The etiology underlying the sparing of the previously irradiated skin maybe due to either decrease in the population of EGFR expressing cells or decrease in the EGFR expression.

We raised the question that "Is it justifiable to use EGFR inhibitors for patients having recurrence in the previously irradiated field?" We may need further research to answer this question which may guide the physicians in choosing appropriate drug in this scenario.

## Introduction

The ErbB or epidermal growth factor family is a family of four structurally related, EGFR/ErbB1/HER1, ErbB2/neu/HER2, ErbB3/HER3, and ErbB4/HER4. ErbB receptors are comprised of an extracellular region or ectodomain, a single transmembrane spanning region, and a cytoplasmic tyrosine kinase domain [1]. Epidermal growth factor receptors (EGFR), upon activation by their respective ligands, undergo a transformation from the inactive monomeric form into an active homo or hetero-dimer. This process stimulates its intrinsic intracellular protein-tyrosine kinase activity [2].

Mutation, amplification, or dysregulation of the EGFR family leads to uncontrolled division and predisposes the individual to cancer development [3]. EGFR over-expression has also been correlated with disease progression, poorer prognosis, and reduced sensitivity to chemotherapy [4]. Inhibiting the EGFR - by directly blocking the extracellular EGFR receptor domain with monoclonal antibodies or by inhibiting the intra-cytoplasmic ATP binding site with tyrosine kinase inhibitors (TKI's) - represents an accepted form of targeted cancer therapy[5].

Data from a large, randomized, phase III study of patients with locally advanced squamous cell carcinoma (SCC) of the head and neck suggests that blockade of the EGFR pathway may improve the efficacy of radiation therapy and improve survival [6]. In this study, EGFR blockade was achieved with the monoclonal antibody Cetuximab (Erbitux). There was no significant difference in the rate of mucositis seen in either treatment arm, but there was a higher incidence of grade 3/4 skin reactions when the combined high dose radiation/Cetuximab was employed. Nonetheless, the addition of Cetuximab was associated with a significant improvement in overall survival (median 54 v 28 months; p = 0.02) compared to radiation alone.

EGFR inhibition, whether with antibodies or TKI, causes a cutaneous rash in almost 70% of patients receiving such therapy; generally it involves the face, neck, and upper chest. The severity of rash has been correlated to progression-free survival in cetuximab and erlotinib treatment and it has been suggested that the rash may be a surrogate marker for efficacy [7]. The severity of the rash peaks during the first 1-2 weeks of therapy, stabilizing in intensity thereafter [8], and it characteristically develops in the following phases:

(a). Sensory disturbance with erythema and edema (week 0-1)

(b). Papulopustular eruption (weeks 1-3)

(c). Crusting (weeks 3-5)

(d). Ending with erythema to telangiectasias (weeks 5-8).

Even if it has resolved or greatly diminished during the second month (weeks 4-6), the erythema and dry skin remain in areas previously dominated by the papulopustular eruption [9].

Here, we report a case of lack of Cetuximab-induced skin rash in an area that had previously been irradiated for SCC and present a brief review of the literature.

## Case Report

A 78-year-old Caucasian male was diagnosed with a well differentiated squamous cell carcinoma (SCC) of the skin over the left ear. This was initially excised and treated with adjuvant radiation treatment using 12 MeV electrons between January and March 2008. An initial dose of 50 Gy was delivered to the external ear and the adjacent lymph node region, followed by a 10 Gy boost to the expanded GTV, and completed with an additional 6 Gy to a residual nodular area on the posterior surface of the ear. He later underwent excision of this nodular area with placement of a skin graft derived from the left supraclavicular area.

In Dec 2008, seven months following completion of his definitive therapy, the patient presented with a palpable swelling in the left upper neck which had been gradually increasing in size for two months (this was in the region that had received 5,000 cGy during the previous course of radiation). A fine needle aspiration biopsy revealed cells consistent with recurrent SCC. Computed tomography (CT) performed for staging showed a solitary 3.1 cm enhancing mass in the left post-auricular region, with infiltration of the left sternocleidomastoid muscle. No other disease was apparent.

Following evaluations in both medical and radiation oncology, the clinical consensus was to proceed with re-irradiation with concurrent Cetuximab. He was therefore treated with 6 MV photons using an intensity modulated radiotherapy (IMRT) technique. Cetuximab was administered in standard fashion concurrently with his radiation therapy.

**Figure 1:**
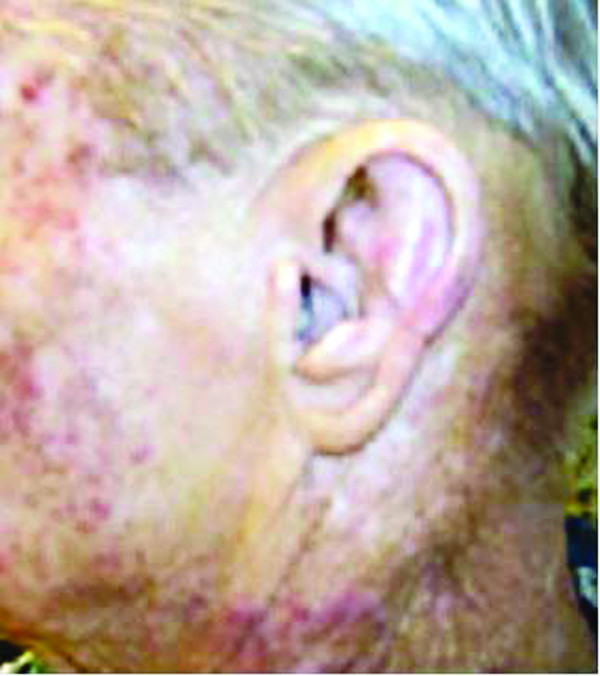
**Left face: Showing lack of skin rash in the previously irradiated area**.

**Figure 2:**
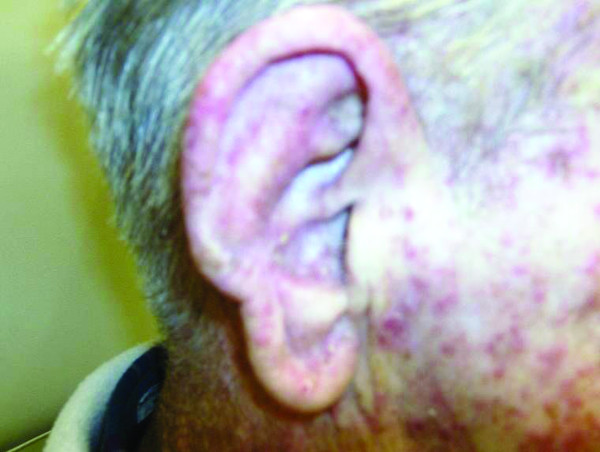
**Right Face: Previous unirradiated side showing typical Cetuximab induced skin rash**.

Approximately two weeks into his treatment, he developed the anticipated papulo-pustular skin lesions on his neck. Surprisingly, however, there was no such skin reaction in the previously irradiated field which shared considerable overlap with the current re-irradiation field (Fig-1 & Fig-2). During follow up appointments the Cetuximab-induced rash seen elsewhere gradually resolved but slight erythema persisted in the area previously covered by the rash.

In May 2009, approximately five months after his radiation treatment, he was found to have a second recurrence in the left neck, corresponding to a level Va lymph node, and he underwent resection of the involved node. In December 2009, he was diagnosed with dermal metastasis involving the left neck, recurrent lymph node involvement corresponding to the area of previous surgical resection in neck, as well as the development of a new node in the left parotid gland. At this point, he was deemed unresectable and was offered palliative chemotherapy.

## Discussion

EGFR is expressed in undifferentiated, proliferating keratinocytes in the basal and suprabasal layers of the epidermis, skin fibroblasts, and in the outer root sheath of the hair follicles [10]. EGFR plays a crucial role in the normal maturation and development of epidermis. Activation of EGFR in the skin causes stimulation of epidermal growth and inhibition of differentiation [11]. Reduction and impairment of functional stem cells, endothelial cell changes, and inflammation constitute the main patho-physiology underlying acute radiation injury [12], whereas microvascular injury from endothelial cell damage and fibrosis, both mediated by TGF-β, is intricately involved in the chronic radiation dermatitis [13]. Cetuximab is a monoclonal antibody inhibitor of the EGFR that has been shown in a large, randomized phase III study to improve survival when delivered concurrently with radiation therapy for advanced head and neck SCC. Its administration is typically associated with a skin rash.

Our review of the literature revealed only a single case series report documenting the lack of a Cetuximab-induced skin rash in the previously radiated field [14]. In this report, Bossi *et al *identified six cases with sparing of skin toxicity induced by cetuximab in previously irradiated areas. The interval between previous radiation and cetuximab therapy ranged from 3 months to 70 months (median 15.5 months). They reviewed the possibility of microvascular injury that may have impeded delivery of the small molecules to their targets.

Despite the single case series report regarding Cetuximab, we identified three case reports documenting sparing of the skin in the previous radiation field in patients treated with erlotinib, a small molecule inhibitor of tyrosine kinase. Again, the interval between the previous radiation treatment and administration erlotinib varied, 9 months after prior treatment with 64 Gy [15], 4 months after 39 Gy [16], and 2 months after 45 Gy [17]. Yalcin et al also biopsied the previously irradiated skin that was devoid of the skin rash and found normal EGFR expression but a conspicuous absence of follicular structures. They hypothesized that the lack of follicular structure in the irradiated skin biopsy was the reason underlying the lack of a skin rash.

Two other case reports have demonstrated a temporal dependence between the timing of erlotinib therapy and the radiation treatment. In a report by Lacouture et al, erlotinib given immediately after radiation treatment resulted in a severe, confluent skin rash over the irradiated area compared to other parts of skin involved with skin rash. It was thought that this phenomenon was secondary to the radio sensitizing properties of the EGFR inhibitors [18]. Gerber *et al *treated a 55 year-old woman with metastatic NSCLC who developed a characteristic papulopustular rash on the face, trunk and upper extremities in response to erlotinib therapy. The rash, however, spared a rectangular area over the lower vertebral column, extending from T8 through T12, where she had received radiation therapy two months previously for osseous metastases. Interestingly, however, two months after erlotinib therapy was initiated and 4 months after radiation treatment was completed, she proceeded to develop the characteristic skin rash in the initially spared area, suggesting a radiation recall type phenomenon [19].

The etiology underlying the sparing of the previously irradiated skin from the expected cetuximab-induced skin rash in our patient is uncertain. In addition to the lack of follicular structures [17], another possible mechanism is the diminished number of EGFR expressing cells after radiation. Alternative hypotheses include stunted receptor function, diminished receptor sensitivity, or impaired transmembrane signaling.

In addition, because the severity of the skin rash has been associated with treatment efficacy [7], one should consider the possibility that the therapy may not be as beneficial in the absence of a skin reaction. Our patient had a recurrence in a previously irradiated area where skin sparing was noted during his re-irradiation. Despite the re-irradiation and subsequent surgical resection he proceeded to develop a recurrence in the same area. He is now deemed unresectable and is undergoing palliative chemotherapy. Although we should be cautious in the use of concurrent epidermal growth factor inhibitors in the radiation treatment of recurrences in previously irradiated areas, further research at the molecular level is necessary to obtain a better understanding of the etiology and variability of the skin toxicity induced in patients undergoing combined therapy.

## Competing interests

The authors declare that they have no competing interests.

## Authors' contributions

MK conceived the idea, did the literature search and prepared the manuscript. SV and SP provided critical review of the manuscript and research guidance. All authors have read and approved the final manuscript.

## Consent

Written informed consent was obtained from the patient for publication of this case report and accompanying images. A copy of the written consent is available for review by the Editor-in-Chief of this journal.
